# Low-dose salinomycin induces anti-leukemic responses in AML and MLL

**DOI:** 10.18632/oncotarget.11866

**Published:** 2016-09-06

**Authors:** Gary D.R. Roulston, Charlotte L. Burt, Laura M.J. Kettyle, Kyle B. Matchett, Heather L. Keenan, Nuala M. Mulgrew, Joanne M. Ramsey, Caoifa Dougan, John McKiernan, Ivan V. Grishagin, Ken I. Mills, Alexander Thompson

**Affiliations:** ^1^ Centre for Cancer Research and Cell Biology, Queen's University Belfast, Belfast, BT9 7AE, Northern Ireland, United Kingdom; ^2^ Cambridge University School of Clinical Medicine, Addenbrooke's Hospital, Cambridge, CB2 0SP, United Kingdom; ^3^ Division of Cancer and Stem Cells, School of Medicine, Wolfson Centre for Stem Cells, Tissue Engineering & Modelling (STEM), University of Nottingham, Nottingham, NG7 2RD, United Kingdom

**Keywords:** drug repurposing, salinomycin, anti-leukemia, gene signature, AML/MLL-rearrangements

## Abstract

Development of anti-cancer drugs towards clinical application is costly and inefficient. Large screens of drugs, efficacious for non-cancer disease, are currently being used to identify candidates for repurposing based on their anti-cancer properties. Here, we show that low-dose salinomycin, a coccidiostat ionophore previously identified in a breast cancer screen, has anti-leukemic efficacy. AML and MLLr cell lines, primary cells and patient samples were sensitive to submicromolar salinomycin. Most strikingly, colony formation of normal hematopoietic cells was unaffected by salinomycin, demonstrating a lack of hemotoxicity at the effective concentrations. Furthermore, salinomycin treatment of primary cells resulted in loss of leukemia repopulation ability following transplantation, as demonstrated by extended recipient survival compared to controls. Bioinformatic analysis of a 17-gene signature identified and validated in primary MLLr cells, uncovered immunomodulatory pathways, hubs and protein interactions as potential transducers of low dose salinomycin treatment. Additionally, increased protein expression of p62/Sqstm1, encoded for by one of the 17 signature genes, demonstrates a role for salinomycin in aggresome/vesicle formation indicative of an autophagic response.

Together, the data support the efficacy of salinomycin as an anti-leukemic at non-hemotoxic concentrations. Further investigation alone or in combination with other therapies is warranted for future clinical trial.

## INTRODUCTION

Drug repurposing (or repositioning) has become a tactic to deploy approved weaponry in the war on cancer, resulting in the development of defined projects [1]. The concept of drug repurposing is borne from a demand to complement and accelerate current drug development strategies, which are often costly, inefficient, [2, 3] and in the case of anti-cancer therapies, can take over 8 years to obtain approval for clinical use [4]. This potential drug development ‘crisis’ is occurring at the same time as increased cancer incidence due in part to an aging society, altered lifestyles and diet in developing countries [5, 6].

Salinomycin is a polyether organic anion that is widely used as a coccidiostatic antibiotic in poultry and ruminant feed. Salinomycin was identified in a chemical screen by Gupta *et al* to target breast cancer stem cells (CSCs) [7], and has subsequently shown anti-neoplastic properties in a range of human cancers, including hematological malignancies (reviewed by Zhou *et al*) [8], though it's activity in acute leukemia remains unexplored. We previously used Connectivity Mapping to identify candidate drugs, including an antibiotic, for repurposing as anti-leukemic agents [9].

As an antibiotic, salinomycin primarily acts as an ionophore, facilitating transportation of cations (e.g. Ca^2+^ and K^+^) across membranes resulting in toxic intracellular levels [10, 11]. As an anti-cancer agent, salinomycin has been reported to activate several pathways including apoptosis in a p53/Caspase/CD95/DC95L/ proteasome independent manner, increase DNA damage, and inhibit Wnt signalling, preferentially in malignant CSCs [12–15]. However, the precise mechanism of action of salinomycin in distinct CSCs remains to be elucidated. One recognized limitation for clinical application of salinomycin is a relatively narrow therapeutic index, giving rise to potential related toxicities [16–18] and potential impairment of normal stem cell function, particularly within the hematopoietic system.

To gain further insight into possible clinical application for hematological malignancies, we examined and demonstrated the anti-leukemic potential of low-dose salinomycin in pre-clinical models of acute myeloid leukemia (AML) and poor prognosis Mixed Lineage Leukemia-rearranged (MLLr) subtypes, whilst concurrently demonstrating negligible toxicity to normal blood cells.

## RESULTS

### Differential sensitivity of human AML cell lines to Salinomycin

**Figure 1:**
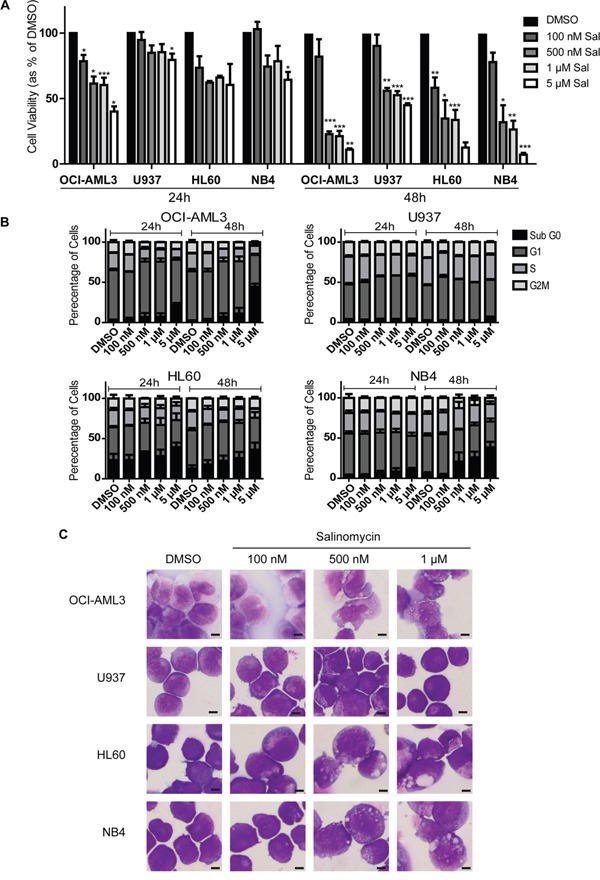
Differential sensitivity of human AML cell lines to salinomycin **A.** Bar graphs showing decreased viability in AML cell lines over time following salinomycin treatment at the indicated dosage compared to 0.01% DMSO vehicle control. **B.** Bar graphs showing altered cell cycle (Propidium Iodide staining) in AML cell lines over time following salinomycin treatment at the indicated dosage or 0.01% DMSO vehicle control. **C.** Representative morphology images of AML cell lines treated for 48 hours with salinomycin at the indicated dosages or 0.01% DMSO. Mean values ± S.E.M. of biological replicates (n=3) are plotted throughout. ***P < 0.001, **P < 0.01, *P < 0.05, *t*-test. Scale bar = 4 μm.

AML cell lines demonstrated reduced cell viability following salinomycin treatment (100 nM - 5 μM), with OCI-AML3 cells demonstrating greatest and U937 cells least sensitivity, particularly at the 24 hour time point (Figure 1A and Supplementary Table S1). Reduced cell viability was associated with an accumulation of cells in the Sub G_0_ phase of the cell cycle as determined by direct DNA staining (Figure 1B) with only limited related changes in Caspase activity (Supplementary Figure S1, upper panel). Although U937 cells demonstrated little increase in the Sub G_0_ population, some alteration in cell cycle progression, primarily reduction of cells in S-phase, was observed.

To further examine the effects of salinomycin, AML cells were exposed to low concentrations (≤1 μM) for 48 hours and examined microscopically. Three of the cell lines demonstrated significant alterations in morphology in a dose-dependent manner, ranging from loss of nuclear shape at low dose exposure (100 nM, HL60) to the formation of cytoplasmic vesicles at higher dosages (500 nM, OCI-AML3, HL60 and NB4). Consistent with the cell viability and cell cycle assays, U937 cells demonstrated no morphological changes compared to DMSO controls (Figure 1C).

### Salinomycin alters growth and morphology of MLLr cell lines

**Figure 2:**
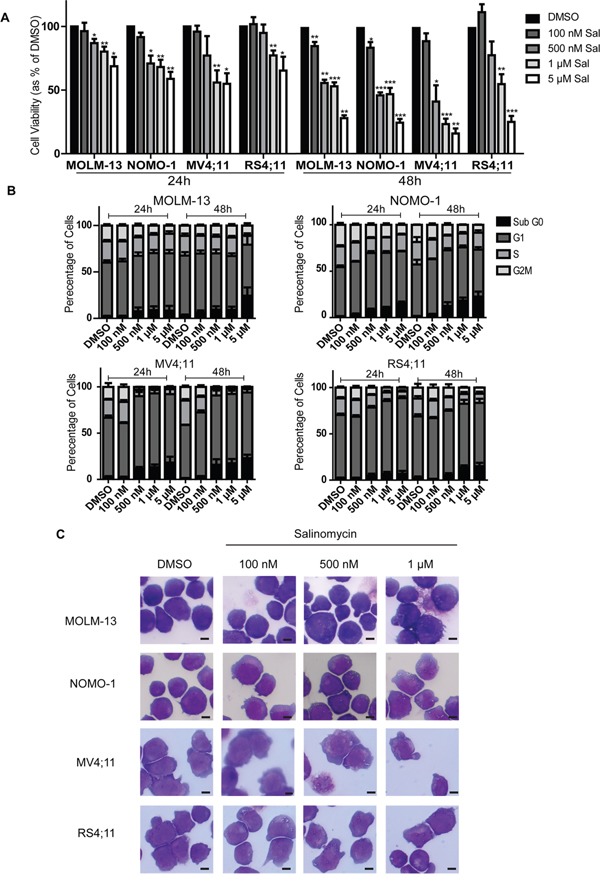
Salinomycin alters growth and morphology of MLLr cell lines **A.** Bar graphs showing decreased viability in MLLr cell lines over time following salinomycin treatment at the indicated dosage compared to 0.01% DMSO vehicle control. **B.** Bar graphs showing altered cell cycle (Propidium Iodide staining) in MLLr cell lines over time following salinomycin treatment at the indicated dosage or 0.01% DMSO vehicle control. **C.** Representative morphology images of MLLr cell lines treated for 48 hours with salinomycin at the indicated dosages or 0.01% DMSO. Mean values ± S.E.M. of biological replicates (n=3) are plotted throughout. ***P < 0.001, **P < 0.01, *P < 0.05, *t-*test. Scale bar = 4 μm.

All four MLLr cell lines were sensitive to salinomycin over the same dose and time used for the AML cell lines, indicative of a biological response (Figure 2A and Supplementary Table S1). Increases in Sub G_0_ were associated with decreases in S-phase populations (Figure 2B and Supplementary Figure S2 upper), and increased Annexin V expression (Supplementary Figure S2) was associated with limited change in Caspase activity, restricted to 1 μM treatment of MV4;11 cells (Supplementary Figure S1, lower panel). Microscopic examination showed similar morphological changes as observed for AML cells, including the presence of vesicles in all MLLr cell lines following 500 nM salinomycin treatments (Figure 2C). Overt vesicle formation in three of the MLLr cell lines examined (MOLM-13, NOMO-1 and RS4;11) was observed even at 100 nM salinomycin compared to vehicle controls, indicating increased sensitivity in these cell lines.

### Salinomycin-sensitive primary murine AML and MLLr leukemia cell lines

**Figure 3:**
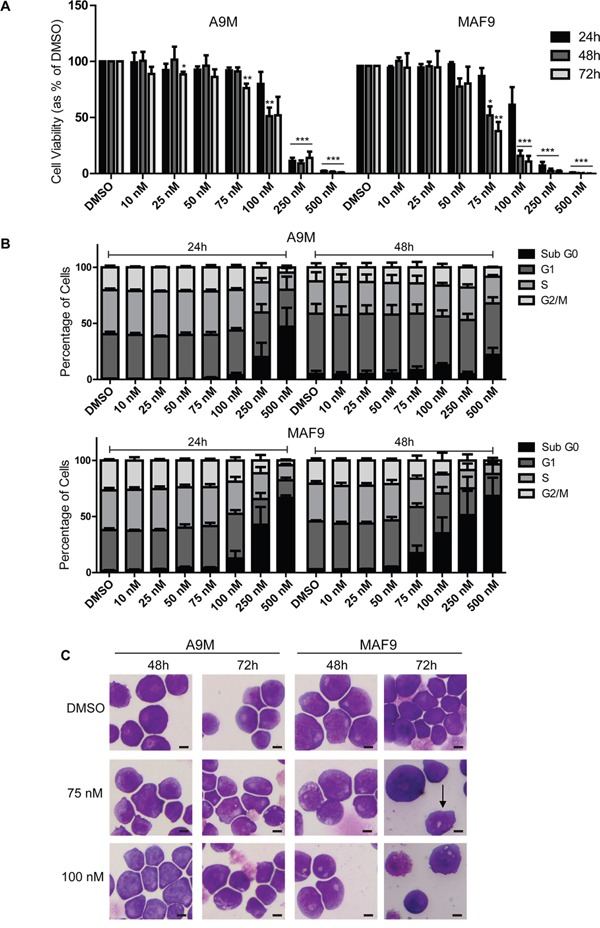
Salinomycin-sensitive primary murine AML and MLLr leukemia cell lines **A.** Bar graphs showing decreased viability in A9M and MAF9 primary leukemia cell lines over time following salinomycin treatment at the indicated dosage compared to 0.01% DMSO vehicle control. **B.** Bar graphs showing altered cell cycle (Propidium Iodide staining) in A9M and MAF9 primary leukaemia cell lines over time following salinomycin treatment at the indicated dosage or 0.01% DMSO vehicle control. **C.** Representative morphology images of A9M and MAF9 primary leukaemia cell lines treated for 48 and 72 hours with salinomycin at the indicated dosages or 0.01% DMSO. Band neutrophil is highlighted with an arrow. Mean values ± S.E.M. of biological replicates (n=4) are plotted throughout. ***P < 0.001, **P < 0.01, *P < 0.05, *t-*test. Scale bar = 4 μm.

To further examine the potential anti-leukemic effect of salinomycin, studies were extended into two growth factor-dependent primary murine cell lines A9M and MAF9, generated by retroviral transduction of *HOXA9-ires-Meis1*(A9M) or *MLL-AF9* (MAF9) into primary haematopoietic cells, as previously reported [9], followed by serial replating in methylcellulose. Due to anticipated increased sensitivity in the primary cells, an extended lower dose range (10 - 500 nM) was used. Both primary cell lines demonstrated reduced cell viability at 250 nM and 500 nM salinomycin at both early (24 hour) and late (72 hour) time points (Figure 3A). Lower dose salinomycin (75 nM and 100 nM) resulted in differential time and cell line response, with the MAF9 cells demonstrating greater sensitivity and lower estimated IC50s (Supplementary Figure S3). The decreased cell viability was matched with dramatic changes in cell cycle, in particular increased Sub G_0_ and decreased S and G2M populations at higher doses (Figure 3B). Consistent with the cell viability assays, cell cycle responses were more striking in MAF9 (lower panel) than A9M cells (upper panel). Since higher doses of salinomycin resulted in significant cell loss, cell morphology analysis in A9M and MAF9 cells was restricted to lower concentrations (75 nM and 100 nM) for up to 72 hours. Morphological changes, including the presence of vesicles, were observed for both cell lines in a time and dose-dependent manner. Interestingly, MAF9 cells also demonstrated the presence of band neutrophils at the 72 hour time point (75 nM), indicative of differentiation (Figure 3C).

### Salinomycin-induced differential gene expression

**Figure 4:**
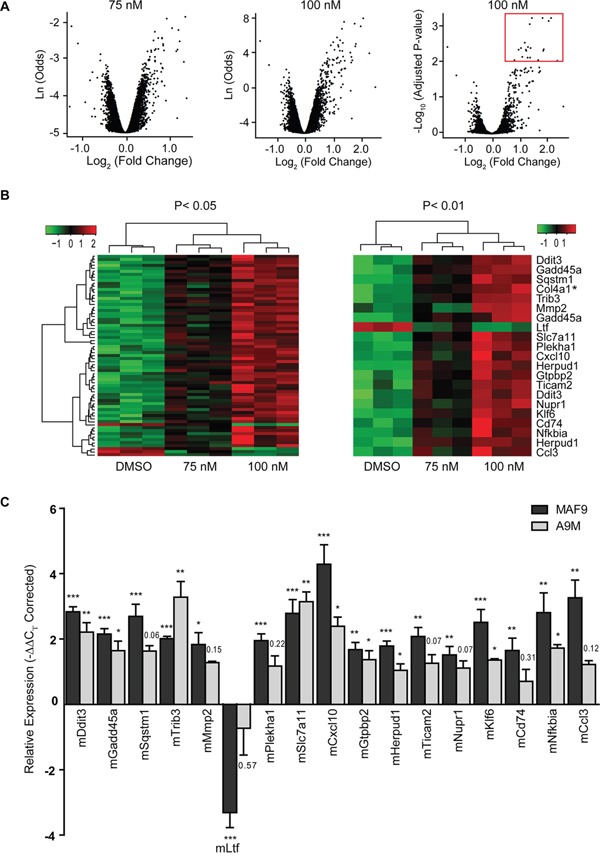
Differential gene expression in treated MAF9 cells **A.** Volcano plots of significance for each of 24,326 probes from salinomycin (75 nM and 100 nM) treated MAF9 cells in the form of B-statistic (log-odds) or negative log_10_ (Adjusted P-value) versus log_2_ (fold change) compared to 0.01% DMSO. Ultimately only genes with −log_10_ (Adjusted P-value) > 2 and log_2_ (fold change) > 0.5 were considered (marked by a red box). **B.** Hierarchical agglomerative clustered heatmaps of samples and transcripts induced or repressed at log_2_ (fold change) of 0.5 or more (P ≤ 0.05) (left panel) were sorted in ascending order (n=18 genes) by adjusted P-value to P ≤ 0.01 (right panel). Hierarchical clustering was based on a Euclidean distance and complete linkage as distance and linkage methods. **C.** Bar graph demonstrating validated expression of 17 of the 18 genes of interest as measured by real-time qRT-PCR. Mean relative expression (−ΔΔC_T_) values ± S.E.M of biological replicates (n=4) were plotted. ***P < 0.001, **P < 0.01, *P < 0.05, *t-*test.

Illumina BeadArray based gene expression profiling was carried out following low dose (75 nM and 100 nM) salinomycin treatment of MAF9 cells, the most sensitive primary mouse cell line examined (Supplementary Table S2). Overall output was characterized by plotting significance in the form of B-statistic (log-odds) or negative log_10_(Adjusted P-value) versus log_2_ expression (fold change) for each gene. Ultimately, only genes with −log_10_(Adjusted P-value) > 2 and log_2_(fold change) > 0.5 were considered (Figure 4A). All samples and transcripts induced or repressed at log_2_ (fold change) of 0.5 or more (P ≤ 0.05) were subjected to hierarchical agglomerative clustering by treatment with salinomycin (Figure 4B, left panel). Clustering was based on a Euclidean distance and complete linkage as distance and linkage methods. Transcripts induced or repressed at log_2_(fold change) of 0.5 or more (P ≤ 0.01) were sorted in ascending order by adjusted P-value. Twenty one probes, representing 18 genes, were identified for further evaluation (Figure 4B, right panel, Supplementary Table S3).

Col4a1 was excluded due to insufficient expression (C_T_ > 35) and the remaining 17 genes were validated with high correlation (r^2^ = 0.901) to the array outputs for MAF9 (Supplementary Figure S4). All 17 genes were significantly differentially expressed (P < 0.05) in the MAF9 cells compared to control (Figure 4C) with *lactotransferrin* being the only gene with reduced expression following salinomycin treatment. qRT-PCR analysis of the salinomycin 17-gene signature was extended to A9M cells and over 50% (9/17) of the genes demonstrated differential expression to a significance level (P < 0.05).

The salinomycin 17-gene signature was further used to interrogate bioinformatic databases. Submission of the gene signature to GeneMania [19], a large association-based database, identified functional networks, including positive regulation of immune response and protein serine/threonine kinase activity, based primarily on co-expression with FDR values < 10^−5^ (Supplementary Figure S5). Submission of the translated 17-gene signature to the protein-protein interaction network STRING [20] identified three primary hubs (NF-kB, chemokine and DNA repair) centrally connected by Tnf, Mapk14 and Akt1 (Supplementary Figure S6A). Application of the signature to DAVID (The Database for Annotation, Visualization and Integrated Discovery) [21, 22] analysis identified the toll-like pathway (KEGG) with significance (P = 0.0063 corrected by the Benjamini-Hochberg method; Supplementary Figure S6B). Together, these analyses identified association of salinomycin treatment with primary immunomodulatory pathways.

### Induction of sequestosome-1(p62) positive aggresomes/vesicles

**Figure 5:**
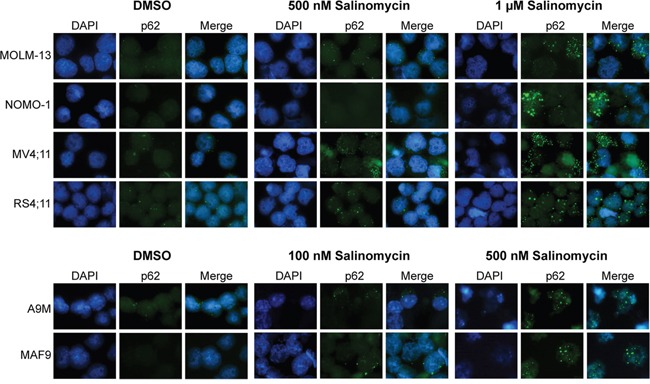
Induction of Sequestosome-1(p62) positive aggresomes/vesicles Combined immunofluorescence staining of SQSTM1/p62 with Alexa Fluor and DAPI depicting accumulation of cytoplasmic aggresomes/vesicles in MLLr, A9M and MAF9 cells treated with salinomycin for 48 hours at the indicated dosages or 0.01% DMSO. Representative images were taken using an x60 objective.

Five of the genes identified as part of the salinomycin-induced signature in MAF9 cells, including sequestosome-1 (*Sqstm1/p62*), were highly significantly upregulated (P < 0.001) compared to control (Supplementary Table S3). Sqstm1 is associated with the formation of polyubiquitin-containing aggresome-like induced structures (ALIS), which resemble the vesicles observed in the salinomycin-treated leukemic cell lines (Figures 1C, 2C and 3C). To examine this further, human MLLr and both primary cell lines were treated with salinomycin and probed for expression of Sqstm1/p62. Treated cells showed cytoplasmic restricted Sqstm1/p62 positivity, by co-immunofluorescence and DAPI staining, in a dose-dependent manner (Figure 5). Quantitative scoring demonstrated a marked increase in the proportion of salinomycin treated Sqstm1/p62 positive cells compared to vehicle control (Supplementary Figure S7) associated with modest increased expression of Atg7 and Beclin-1, particularly in the more sensitive MAF9 cells, as determined by western blot analysis (Supplementary Figure S8). Together, these data indicate activation of an autophagic response by AML/MLLr cells to low-dose salinomycin treatment.

### Anti-leukemic effects of salinomycin in primary cells

**Figure 6:**
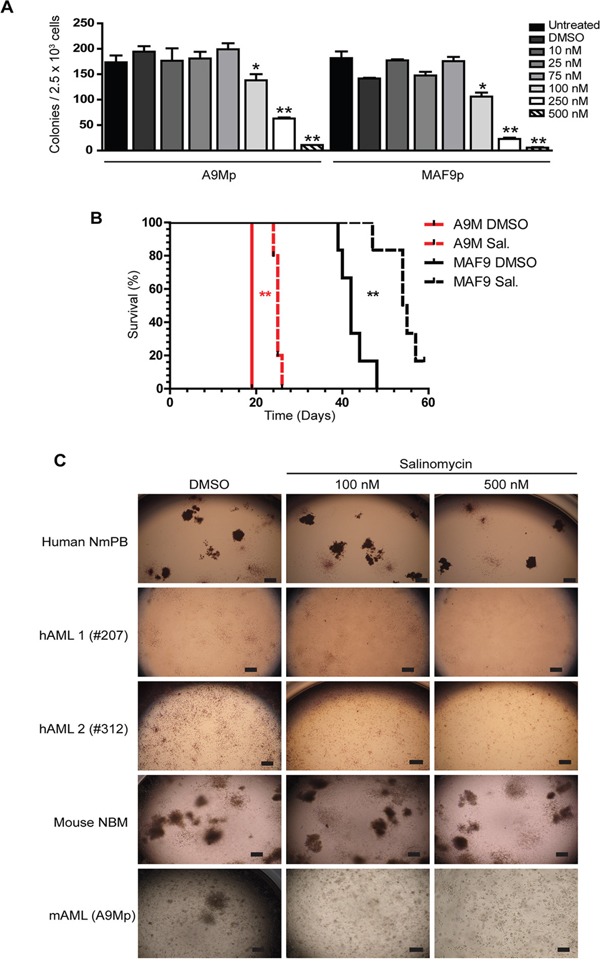
Anti-leukemic effects of salinomycin in primary cells **A.** Bar graphs showing decreased myeloid colony formation in untreated A9Mp and MAF9p primary mouse leukemia cells or following salinomycin treatment at the indicated dosage or 0.01% DMSO vehicle control. **B.** Kaplan Meier plots demonstrate extended survival in recipient mice (n=5 per group) following transplantation of salinomycin treated A9Mp or MAF9p primary leukemia cells compared to untreated or 0.01% DMSO vehicle controls. P-values, Log-rank (Mantel-Cox) Test. **C.** Representative images of colony formation of human normal mobilized peripheral blood (NmPB), patient samples hAML-1(#207), hAML-2 (#312), mouse normal bone marrow (NBM) and mouse AML (A9Mp) following salinomycin treatment at the indicated dosage or 0.01% DMSO vehicle control. Colonies were stained with 1 mg/ml p-iodonitrotetrazolium violet for 16 hours and images captured at x2 using an inverted microscope. Scale bar = 4 μm.

Primary mouse models and patient samples were next used to examine the anti-leukemic activity of salinomycin. Normal bone marrow (mouse) and donor derived mobilized peripheral blood (human) cells served as controls to examine potential toxicity to normal hematopoietic cells. As for the generated mouse cell lines, a range of low dose salinomycin (10 nM to 500 nM) was used to treat the corresponding primary leukemia cells (A9Mp and MAF9p, respectively). Myeloid colony formation for both primary leukemias was impaired in a dose dependent manner compared to controls (Figure 6A). To examine effects on leukemia maintenance, vehicle control or salinomycin treated primary leukemias were transplanted into recipient mice. Extension in survival was observed for both the early onset A9Mp (P = 0.0027) and late onset MAF9p cells (P = 0.002) treated with 500 nM salinomycin compared to controls (Figure 6B). The short extension in survival (~ 6 days) for the more aggressive A9M compared to the more sensitive MAF9 leukemias may reflect differences in cell of origin or repopulating cell numbers. Transplantation of salinomycin treated normal bone marrow cells had no deleterious effect on recipient mice within the time of evaluation (data not shown). Colony formation of mouse normal bone marrow (NBM) or normal mobilized peripheral blood (NMPB) was not markedly affected by salinomycin treatment (even at 500 nM), whereas the ability to form colonies was impaired by 100 nM and inhibited by 500 nM salinomycin treatment in both human (hAML1-3) and mouse (A9Mp, MAF9p) primary leukemias (Figure 6C). Interestingly, both mouse and patient derived leukemia cells appear to retain metabolic activity (as seen by incorporation of the INT stain) but the ability to form small clusters or colonies is impaired.

## DISCUSSION

Novel approaches to drug discovery for hematological malignancies are essential to take advantage of advances made in molecular classification over the last decade. Drug repurposing represents an attractive and economical option in which a novel application for an existing drug is indicated e.g. Metformin, the anti-type 2 diabetes mellitus drug, was recently found to have anti-neoplastic effects in several tissues including gynecological cancers [23]. Salinomycin is a polyether antibiotic with promising anti-cancer properties [7, 8, 12, 15], albeit at the micromole level with associated potential toxicities [24, 25]. A specific study on its potential clinical use in the acute leukemia context has not been reported.

Here, we investigated the anti-leukemic effects of low dose (sub-micromole) salinomycin in human and murine pre-clinical models, of AML and MLLr, including primary leukemia cells. Low dose salinomycin treatment resulted in reduced cell viability, increased apoptosis, vesicle formation and differentiation. A greater sensitivity was observed throughout for MLLr cells. The time and dose-dependent manner of the salinomycin effects indicated a biological response. Gene expression profiling and validation of the most sensitive murine MLLr cells revealed a salinomycin induced 17-gene signature, subsequently analyzed in the murine AML cells. Two of the genes verified in both primary cell lines, *Klf6* and *Ddit3*, are markers of oxidative stress recently reported as upregulated by salinomycin in prostate cancer cells [26]. An additional gene identified, *p62*/*Sqstm1*, is required for aggresome formation linked to degradation of ubiquitinated proteins by autophagy [27]. Further analysis confirmed increased SQSTM1-positive aggresome formation and increased expression of Atg7 and Beclin-1 in salinomycin treated leukemic cells. Salinomycin has been reported to induce autophagy in other cancers [28–30]. However, whether this response is cancer cell protective or destructive requires further investigation. SQSTM1 was recently shown to shuttle polyubiquitinated proteins to nuclear promyelocytic leukemia bodies [31], and a specific role in this context may warrant further examination.

Submission of the gene signature to online bioinformatics databases identified several pathways and network hubs including: positive regulation of immune response and serine/threonine kinase activity (GeneMania); NF-kB, DNA-damage and chemokine hubs (STRING) and the Toll-like receptor pathway (DAVID). Together these analyses indicate activation of an immunomodulatory response by salinomycin treatment. Immunomodulation is well established as both a cellular and molecular therapy in hematological malignancies, including leukemia [32–34]. Specifically, activation of Toll-like receptor pathways promotes differentiation and growth inhibition in AML [35] and demonstrates efficacy in combination with anti-multiple myeloma agents including bortezomib, lenalidomide, melphalan or vorinostat [36]. Activation of an immune response may also reflect inherent inhibition of these pathways in MLLr and HOXA9-Meis-induced malignant cells, as recently reported for MN1 [37], and highlight a potential therapeutic role for salinomycin in these specific leukemias. The specific loss of colony formation of primary leukemia cells and associated loss of repopulating ability *in vivo* supports the reactivation of differentiation pathways by low dose salinomycin treatment. The fact that cells remain metabolically active yet incapable of forming colonies may also reflect subtle differences in mitochondrial and biosynthesis requirements for AML cells that are susceptible to antibiotics and can be exploited [37–42].

Indeed, association with chemotherapy [43] or combination with experimental therapies, including histone deacetylase inhibitors, in the form of anticancer kits that exploit cytotoxic and neo-protection [44], as recently reported for solid tumours [45, 46], may provide the best method to exploit the observed anti-leukemic effects of salinomycin.

Together, the data demonstrates efficacy of non-toxic low dose salinomycin treatment against poor prognostic MLLr and AML and provides insights into the underlying molecular mechanisms. Further investigation of the mechanism and *in vivo* use of low dose salinomycin as a monotherapy or in combination with either chemotherapy or epigenetic modifiers is warranted, to provide the basis for future clinical application.

## MATERIALS AND METHODS

### Human cell lines, murine primary cell lines and leukemias and patient samples

Established, well-characterized and authenticated (DSMZ-2015), mycoplasma free human AML (OCI-AML3, U937, HL60, NB4, and K562), t(9;11) MLLr (MOLM-13 and NOMO-1) and t(4;11) MLLr (MV4;11, and RS4;11) cell lines were maintained at 37°C with 5% CO_2_ in Roswell Park Memorial Institute (RPMI) 1640 medium supplemented with 10% Fetal Calf Serum (FCS), 2 mM L-glutamine, 100 U/ml penicillin and 100 μg/ml streptomycin (RPMI-10; all Life Technologies, Paisley, UK). Low passage numbers of ATCC or DSMZ-authenticated stocks were used throughout. Murine leukemia cell lines were generated by retroviral transduction of lineage depleted (Lin^−^; Stem Cell Technologies, Vancouver, Canada) mouse primary bone marrow cells with MSCV-HOXA9-ires-Meis1 (A9M) and MSCV-MLL-AF9 (MAF9), following serial replating (x3) in MethoCult™ GF M3434 medium (Stem Cell Technologies). Single colonies were selected and expanded for each leukemia, and maintained in RPMI-10 supplemented with cytokine specific conditioned media (5 ng/ml mIL-3, 5 ng/ml mIL-6, 50 ng/ml mSCF and 5 ng/ml mGM-CSF; RPMI-10^+^) for over 2 months to become immortalized, as previously reported [47]. Cell lines generated were maintained at a density between 1-5×10^5^ cells per ml and seeded every two to three days. Primary leukemias derived from the same retroviral vectors (A9Mp and MAF9p) were also maintained *in vivo*. In accordance with the tenets of the Declaration of Helsinki, patient samples and mobilized peripheral blood cells were collected, anonymized and analyzed following informed consent and ethical committee (OREC-NI) approval. Mononuclear cells purified using Ficoll-Paque™ (GE Healthcare BioSciences AB, Uppsala, Sweden) gradient centrifugation, were frozen and stored prior to treatment.

### Animals, vectors and transplantation assays

Male and female congenic donor CD45.1+ (C57Bl6/Pep3b) or (C57Bl/6-Ly5.1) and recipient CD45.2+ (C57Bl/6J) mice (10-16 weeks old) were bred and maintained in a specific pathogen-free (SPF) animal facility (BSU-QUB). Animal handling followed the guidelines of the UK Animals (Scientific Procedures) Act 1986. Experimental procedures were approved by the Animal Welfare Ethical Review Board (AWERB), Queen's University Belfast. Age-matched, randomized, animals were used throughout. Generation of vesicular stomatitis virus-pseudotyped retroviruses, infection of hematopoietic cells and transplantation into mice were performed as previously described [48].

### Cell viability, cell cycle and morphology

Human and murine cell lines were treated for up to 72 hours in RPMI-10 or RPMI-10^+^ respectively containing either salinomycin (Sigma-Aldrich, Gillingham, UK) at the stated concentration or 0.01% DMSO as vehicle control. Cell viability was determined by CellTiter-Glo^®^ (Promega, Madison, WI, USA). For cell cycle analysis, samples were fixed in 70% ethanol and DNA stained by Propidium Iodide (Sigma-Aldrich). DNA content was measured with an LSR II flow cytometer and analyzed using FACS DivaTM (BD Biosciences, Franklin Lakes, NJ, USA) or Cyflogic software (CyFlo Ltd, Turku, Finland). Cells were pelleted, re-suspended in 200 μl phosphate buffered saline (PBS), cytospun (28 x g) for 5 minutes (Cytospin3, Thermo Shandon, Runcorn, UK) and stained with Modified Wright's stain, for morphology analysis.

### Gene expression profiling

Total RNA was isolated using Trizol^®^ (Life Technologies), and its quality was assessed using Agilent RNA 6000 Nano chip and Agilent 2100 Bioanalyzer (Agilent Technologies, Santa Clara, CA) according to the manufacturer's instructions. RNA integrity (RIN) values of >9 were obtained for all samples prior to application to the Mouse Ref-8 expression v.2 BeadArray (Illumina, San Diego, CA), according to the manufacturer's instructions. Briefly, 550 ng of each RNA sample was reverse transcribed then *in vitro* transcribed to generate biotinylated cRNAs. Aliquots of labelled cRNAs (750 ng) were then hybridized to the BeadArray for 16-18 hours at 58°C. Signal detection was achieved using Amersham fluorolink streptavidin-Cy3 (GE Healthcare Bio-Sciences, Little Chalfont, UK) and scanned images obtained using the Illumina BeadArray confocal scanner. Bead level data of biological triplicates were transformed to log_2_ scale, normalized (quantile method) using *beadarray* R package [49] and annotated (Gene Symbols, Names, Entrez IDs, and Probe Quality Grades) using *illuminaMousev2.db* R package [50]. Probes with poor quality grades were removed. Differential expression was then assessed by linear regression followed by parametric empirical Bayes analysis using *limma* R package [51] and output obtained by plotting significance versus log_2_ expression (fold change). Genes with expression ratios above log_2_(0.5)-fold between treatments and control (P < 0.05 and P < 0.01) were identified. False discovery rates (FDR) were controlled using the Benjamini-Hochberg algorithm. Hierarchical clustering with complete linkage and Euclidean distance, as a measure of similarity, was performed.

### qRT-PCR validation of beadarray data

Total RNA isolated from salinomycin or vehicle treated MAF9 and A9M cells was converted to cDNA [9]. Expression of 18 candidate genes with Peptidylprolyl isomerase A 1 (*Ppia1*) as endogenous control was quantified using technical duplicates of biological replicates (n=4). Genes and corresponding primers are listed (Supplementary Table S4). Assays were performed with FastStart Universal SYBR Green Master Mix (Roche) on the Applied Biosystems 7900HT Fast Real-Time PCR System using Sequence Detection System (SDS v2.3) software as per manufacturers’ protocols. Technical duplicates with standard deviations <0.6 C_T_ were averaged, else a single value closest to other biological replicates was used.

Data were processed as reported previously [52]. Briefly, mean ΔC_T_ values and standard errors calculated for each control and treatment group were normalized to yield −ΔΔC_T_ (=ΔC_T_^control^ −ΔC_T_^treatment^) values, which effectively constitute log_2_ fold changes in expression of genes of interest in treated samples compared to control.

### Immunofluorescence

Treated and control cells were collected by centrifugation (107 x g), re-suspended in PBS, and 1 × 10^5^ cells cytospun (28 x g) onto glass slides (Cytospin3, Thermo Shandon Ltd., Runcorn, UK). Cells were fixed in 4% Paraformaldehyde (Sigma-Aldrich) for 15 minutes, washed (x 2) in PBS, permeabilized in 0.2% Triton X-100/PBS (Sigma-Aldrich) for 15 minutes, then incubated in 3% Bovine Serum Albumin Fraction V (BSA; Sigma Aldrich) for 30 minutes, all at room temperature. SQSTM1/p62 Alexa Fluor monoclonal antibody (Clone 864807; R&D Systems, Abingdon, UK) was diluted 1:10 in 3% BSA/PBS, as per the manufacturer's instructions, and slides were incubated overnight at 4°C in a humidified chamber. Following primary antibody labelling, slides were washed (x3) in PBS, air-dried, and mounting medium containing 4′, 6-diamidino-2-phenylindole (DAPI) applied prior to coverslip application. Slides were stored at 4°C prior to image capture using a Nikon Eclipse Ti microscope (x60 objective) (Nikon, Surrey, UK).

### Salinomycin treatment of primary leukemias

Fresh primary murine leukemia or rapidly thawed primary AML patient bone marrow and mobilized peripheral blood samples were allowed to recover in RPMI-10^+^ or RPMI-10 respectively. Primary cells were treated with salinomycin or DMSO for 16 hours in liquid culture then plated in methylcellulose. Similarly treated human cells were seeded at 1 × 10^5^/ml in MethoCult™ H4434 Classic (Stem Cell Technologies) and treated mouse leukemias were either seeded at 5 × 10^5^/ml in MethoCult™ GF M3434 medium (Stem Cell Technologies) or directly transplanted (5 × 10^5^ cells) into sub-lethally irradiated (850 cGy) recipient mice. Recipient mice were followed for up to 80 days, or disease endpoint, at which point necropsy was performed.

### Methylcellulose assays, staining and imaging

Methylcellulose cultures were maintained at 37°C with 5% CO_2_ for up to 10 days when colony formation, colony counts, and images were captured and analyzed. Colony staining with 1 mg/ml p-iodonitrotetrazolium violet (INT, Sigma-Aldrich, Gillingham, UK) for 16 hours enabled visualization of colonies and metabolic activity.

### Statistical analysis

Student's t-tests were performed using GraphPad Prism software v5.0 (GraphPad Software, La Jolla, CA, USA). Sample sizes of similar variance (n ≥ 3 for cell lines and n ≥ 5 for animal studies) were selected based on previous experience to provide sufficient statistical power. Means ± SEM were plotted and significance denoted by the value or as * p ≤ 0.05; ** p ≤ 0.01, *** p ≤ 0.001 throughout.

## SUPPLEMENTARY MATERIALS METHODS FIGURES AND TABLES






