# Supplementary material for: Low-dose salinomycin induces anti-leukemic responses in AML and MLL
Source: Oncotarget. 2016 Sep 6;7(45):73448–61. doi: 10.18632/oncotarget.11866 (PMC5341990; doi:10.18632/oncotarget.11866)
Supplement: Supplementary file 1 [file oncotarget-07-73448-s001.pdf]

Low-dose salinomycin induces anti-leukemic responses in AML and MLL

SUPPLEMENTARY MATERIALS AND METHODS

Apoptosis-based assays

Induction of apoptosis was assessed by measurement of caspase 3/7 activity or Annexin V staining. Caspase activity was evaluated using the Caspase-Glo® 3/7 luminescent assay system (Promega, Madison, WI, USA) as per the manufacturer's instructions for suspension cells. Membrane changes associated with the onset of programmed cell death were evaluated using an apoptosis detection kit (Annexin V-FITC protein, PI; eBioscience, Inc., San Diego, CA, USA), as per manufacturer's instructions, and subsequent measurement using a LSR II flow cytometer (BD Biosciences). Histogram plots were obtained and analyzed using Cyflogic software (CyFlo Ltd, Turku, Finland).

Western blot analysis

Total cell lysates were prepared after incubation of leukemic cells in media containing vehicle (0.01% DMSO) or salinomycin at the indicated dosage and time, using an SDS extraction protocol (Cell Signaling Technology, Danvers, MA, http://www.cellsignal.com). Proteins were separated by SDS-PAGE and probed with Atg7 (D12B11) and Beclin-1 (D40C5) at 1:1000 dilutions both from Cell Signaling. Protein loading was assessed using a mouse monoclonal β-actin primary antibody (1:5,000; Sigma-Aldrich, St Louis, MO). Blots were developed with a chemiluminescence detection system (Immobilon Western Chemiluminescent HRP Substrate; MerkMillipore, Billerica, MA), exposed to X-ray film (SLS/MOL 7016, Analab, Lisburn, U.K.) for up to 10 minutes, developed, and images were obtained using an Auto-Chemi Imaging system (UVP, Upland, CA).

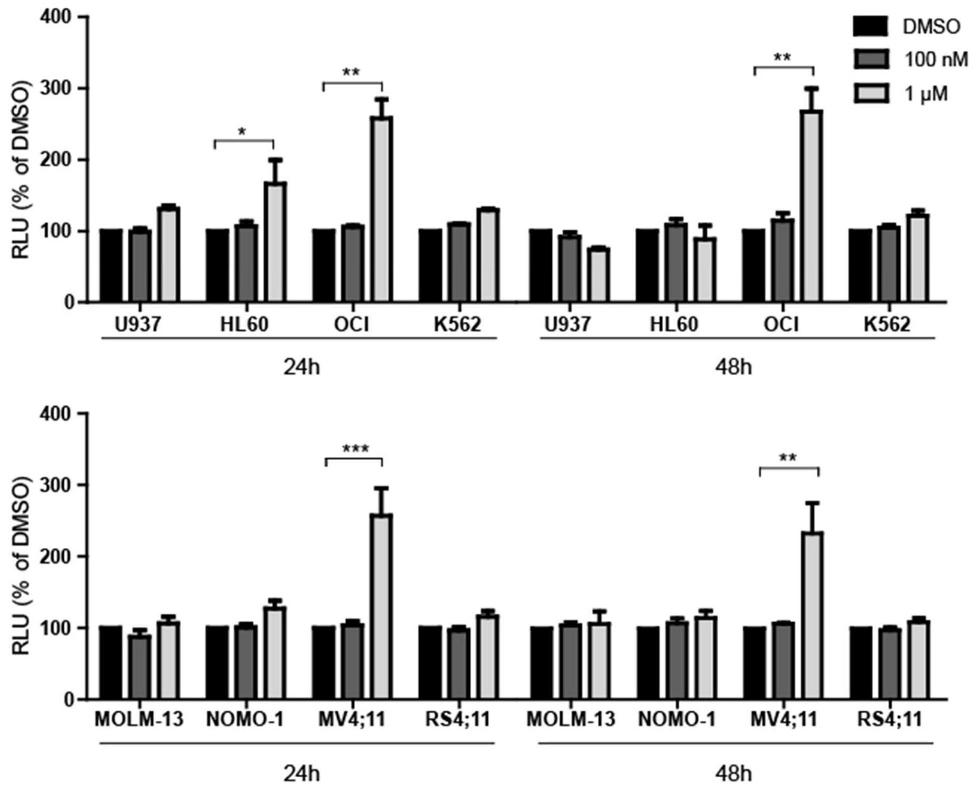

Supplementary Figure S1: Caspase 3/7 activity in AML and MLLr cell lines. Bar graphs showing increased caspase 3/7 activity, as relative luminescence units (RLU) compared to 0.01% DMSO control, following salinomycin treatment (1 μM) is limited to HL60 (24 hours), OCI-AML3 and MV4;11 human cell lines (24 and 48 hours). Mean values ± S.E.M. of biological replicates (n=3) are plotted. ***P < 0.001, **P < 0.01, *P < 0.05, t-test.

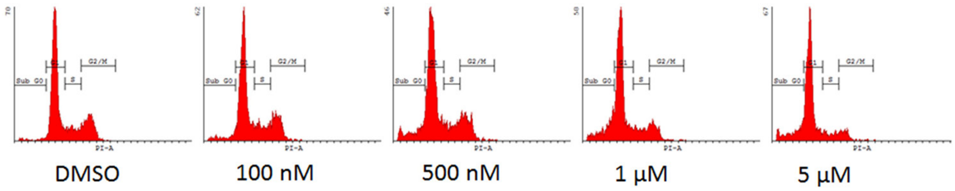

	Sub G0 (%)	G1 (%)	S (%)	G2M (%)
DMSO 24hr	2	63.95	12.4	21.64
100 nM Sal 24hr	4.33	61.52	14.51	19.62
500 nM Sal 24hr	10.65	60.99	11.88	16.41
1 µM Sal 24hr	16.32	61.87	10.7	11.06
5 µM Sal 24hr	15.31	68.96	8.08	7.59

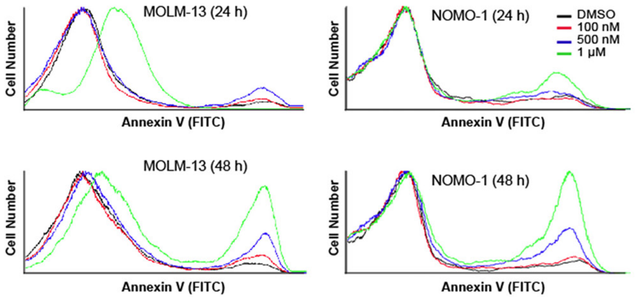

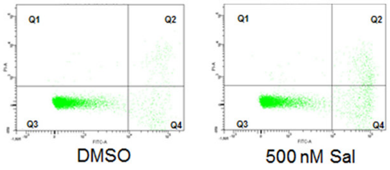

	DMSO	500 nM Sal
Q1 (%)	0.2	0.3
Q2 (%)	1.6	4.4
Q3 (%)	94.9	87.7
Q4 (%)	3.2	7.7

Supplementary Figure S2: Salinomycin increases the Sub G0 fraction and induces Annexin-V expression in MLL-AF9 cell lines. Representative histogram plots following propidium iodide (PI) staining of MOLM-13 cells treated with vehicle (DMSO) or salinomycin for 24 hours at the indicated concentration with associated table depicting percentage of cells in each phase of the cell cycle (upper panel). Overlay histogram plots and representative gating strategy showing increased Annexin-V (FITC) in salinomycin versus DMSO treated human MLL-AF9 cell lines (lower panel). Representative of biological replicates (n=3).

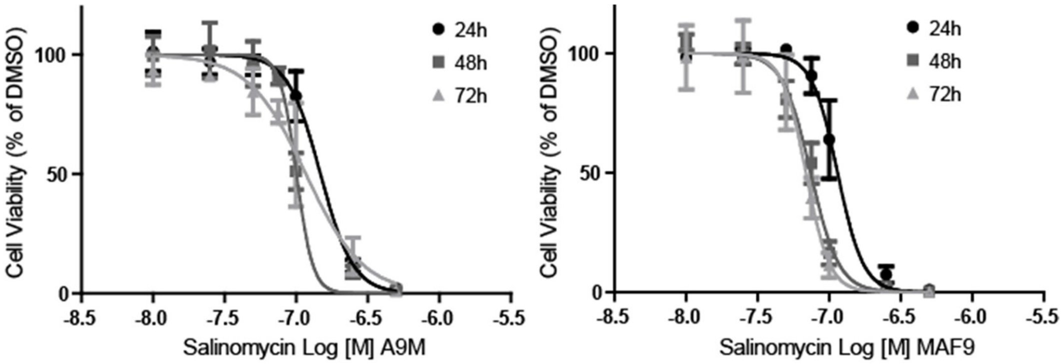

Cell Line	IC50 (nM)		
	24 hour	48 hour	72 hour
A9M	148	101	117
MAF9	114	74	69

Supplementary Figure S3: Dose response curves of salinomycin treated primary murine leukaemia cell lines. Dose-response curves plotting cell viability, compared to 0.01% DMSO, against log-transformed salinomycin concentration after 24, 48, and 72 hours. Estimated IC50 values (tabulated above) were obtained from dose-response curves. The 48 hour IC50 values formed the basis for the treatment regimen of MAF9 cells prior to Illumina BeadArray analysis. Mean values ± S.E.M. of biological replicates (n=3) are plotted.

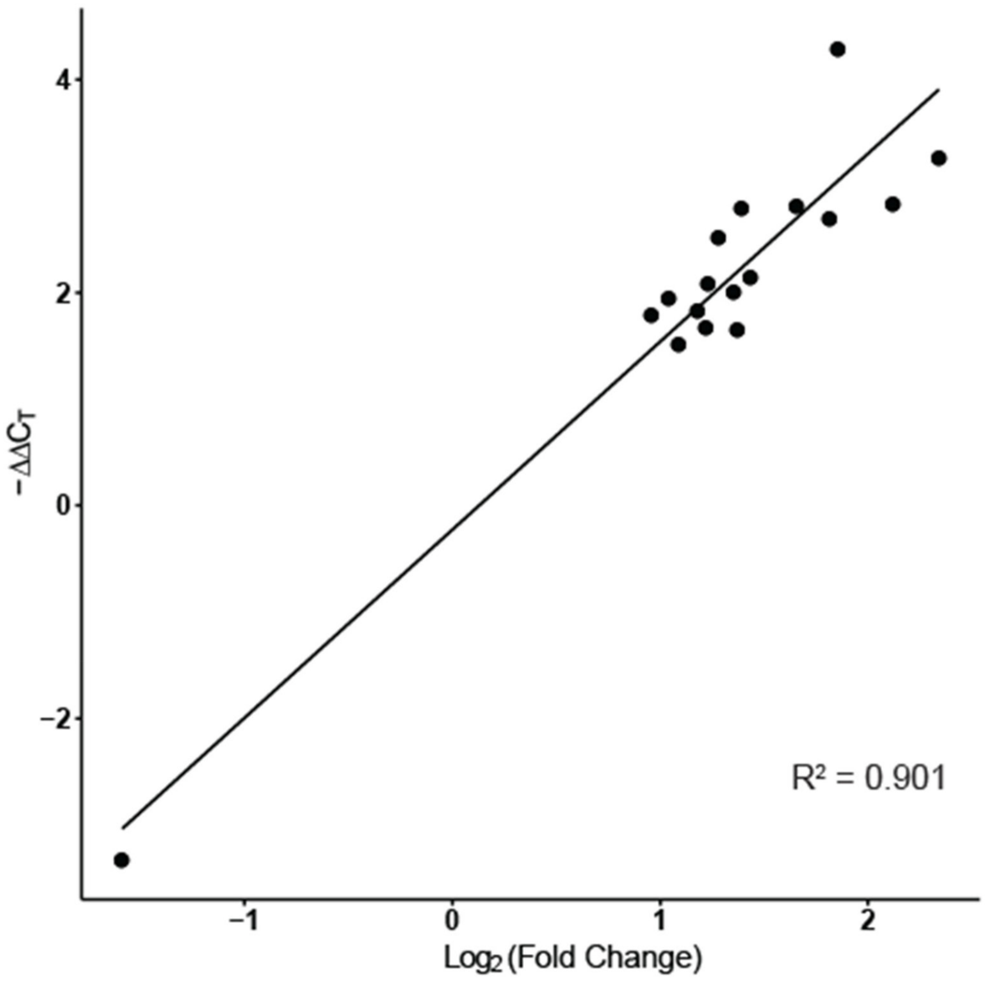

Supplementary Figure S4: Correlation of Illumina BeadArray and qRT-PCR salinomycin 17-gene signature. Scatter graph showing highly significant correlation in expression of the salinomycin 17-gene signature, depicted by a linear regression coefficient ($R^2 = 0.901$) between values obtained from Illumina BeadArray Log_2 (Fold Change) and qRT-PCR $-\Delta\Delta C_T$ platforms.

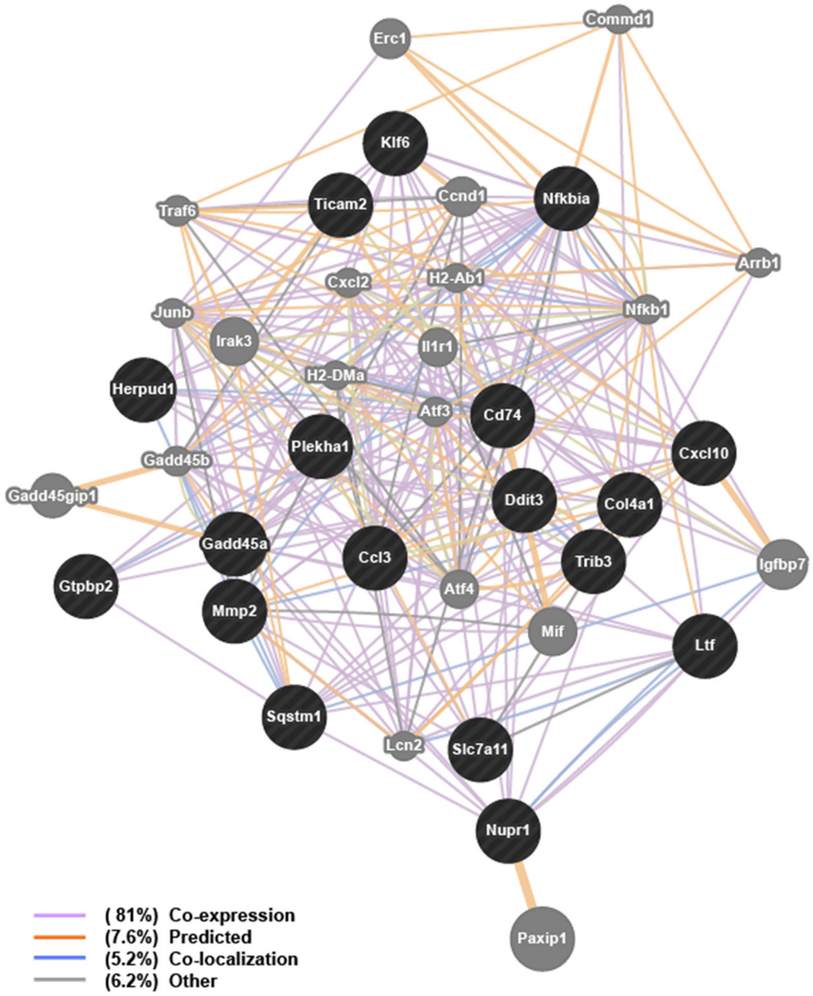

Function	FDR	Coverage
positive regulation of immune response	4.37E-08	11(292)
response to molecule of bacterial origin	1.53E-07	9 (180)
cellular response to biotic stimulus	1.53E-07	8(113)
cellular response to lipopolysaccharide	1.35E-06	7(93)
response to lipopolysaccharide	1.42E-06	8(161)
cellular response to molecule of bacterial origin	1.62E-06	7(101)
positive regulation of defense response	3.19E-06	8(186)
cellular response to lipid	3.74E-06	8(193)
positive regulation of protein serine/threonine kinase activity	5.77E-06	8(207)

Supplementary Figure S5: Functional networks identified by GeneMania. GeneMania identified nine functional networks for the salinomycin 17-gene signature including positive regulation of immune response and protein serine/threonine kinase activity, with FDR $< 10^{-5}$.

A

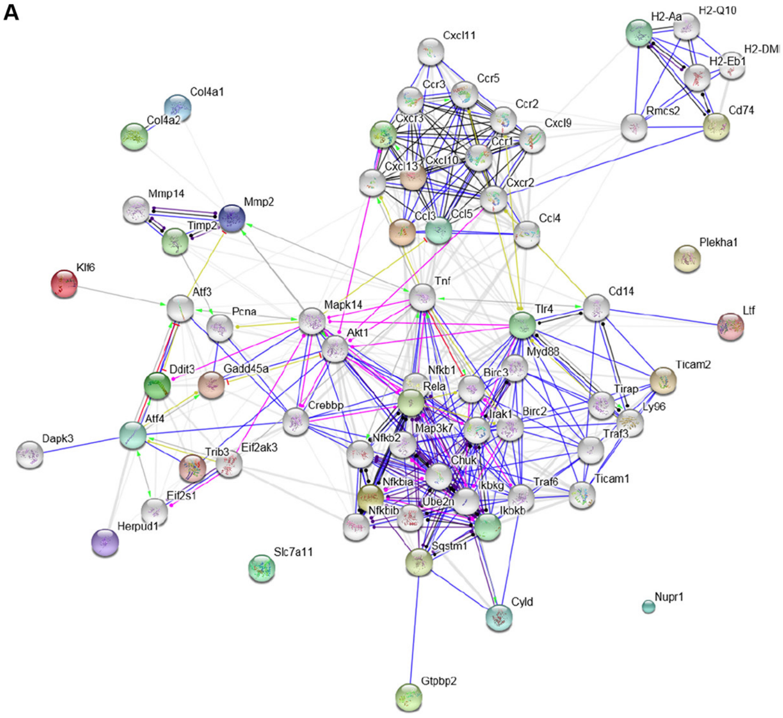

B

Category	Term	Genes	(%)	P-value	Benjamini
KEGG_PATHWAY	Toll-like receptor signaling pathway	4	22	2.90E-04	7.00E-03
GOTERM_BP_FAT	Response to wounding	5	28	5.30E-04	8.10E-02
GOTERM_BP_FAT	Immune response	5	28	1.40E-03	1.10E-01
GOTERM_BP_FAT	Inflammatory response	4	22	2.00E-03	1.20E-01
GOTERM_BP_FAT	Defense response	5	28	1.20E-03	1.20E-01

Supplementary Figure S6: STRING and DAVID analysis of the salinomycin 17-gene signature. A. Submission of the salinomycin 17-gene signature to STRING generated three primary hubs (NFκB, chemokine, and DNA repair) centrally connected by Tnf, Mapk14, and Akt1. **B.** Gene Ontology analysis of the salinomycin 17-gene signature by DAVID identified the Toll-like receptor signaling pathway as being altered in MAF9 cells.

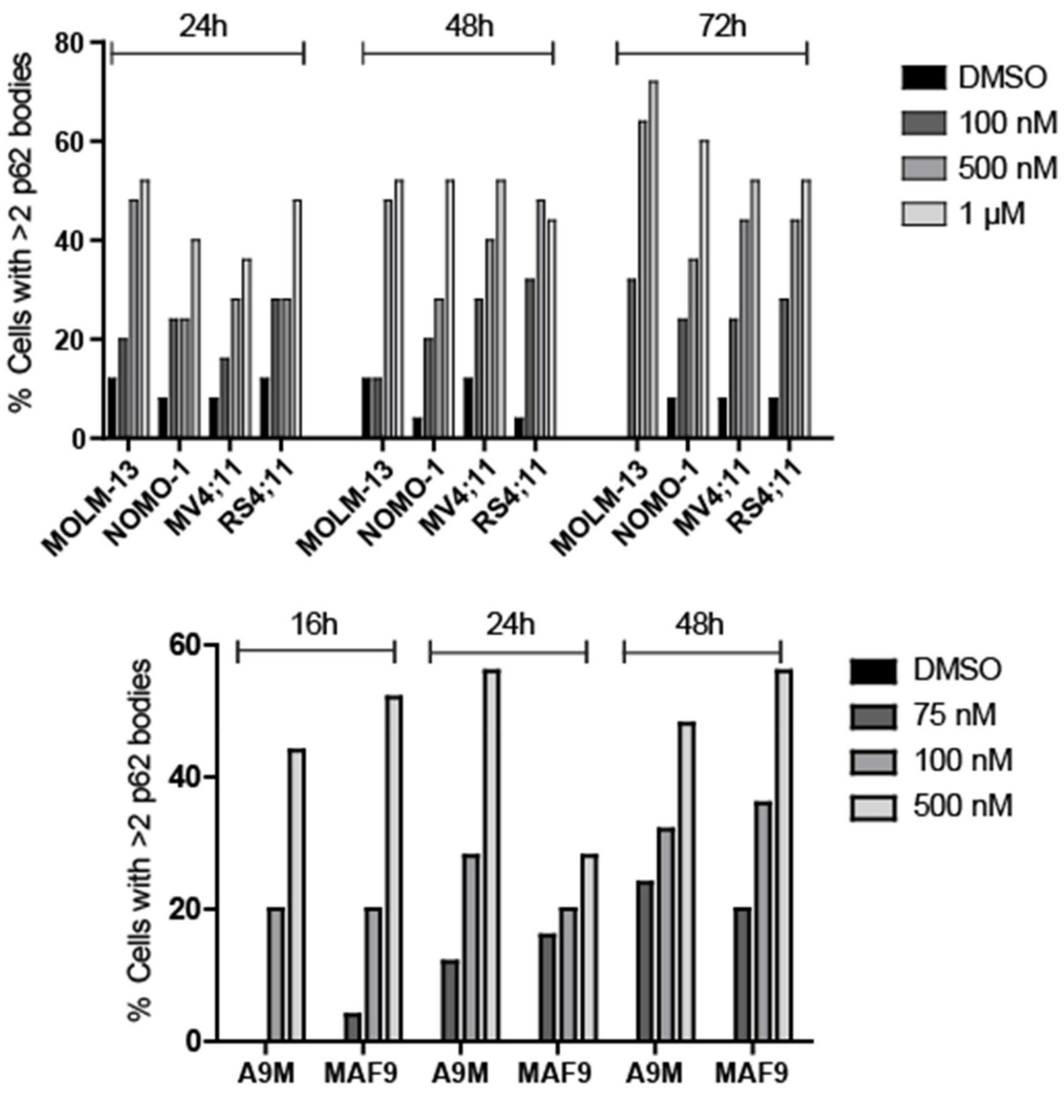

Supplementary Figure S7: Quantitative scoring of p62/SQSTM1 aggresomes in cell lines. Bar graphs showing quantitative scoring of p62/SQSTM1 aggresomes in both human (upper panel) and primary murine (lower panel) cell lines. Twenty-five cells per condition were examined and percentage of cells with greater than two p62/SQSTM1 positive aggresomes calculated and plotted.

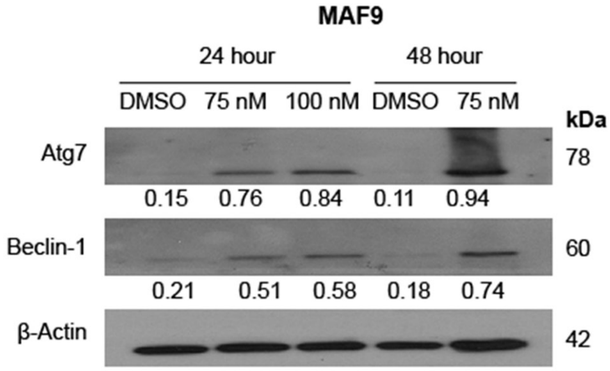

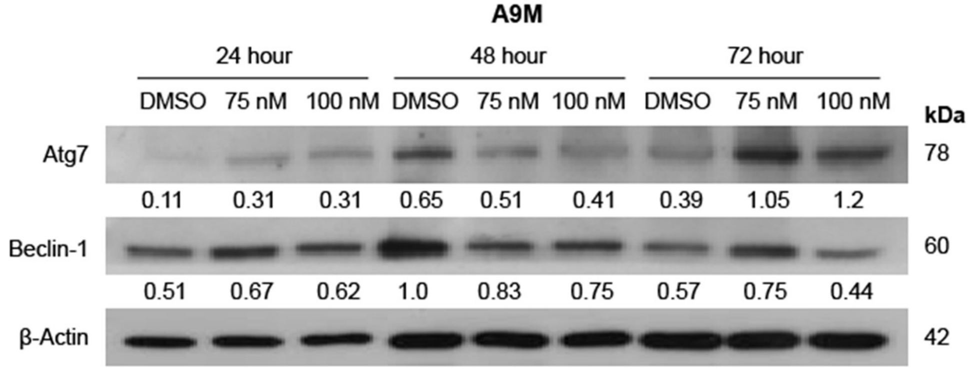

Supplementary Figure S8: Salinomycin-treated A9M and MAF9 cells expressed differential levels of autophagy-associated proteins. Representative western blot analysis demonstrating marked increased expression of the autophagy related proteins Atg-7 and Beclin-1 in salinomycin-treated MAF9 cells (upper panel) and more moderate increase in A9M cells (lower panel) compared to β-actin loading control. Densitometry analysis was performed using Image Studio Lite Version 5.2 (LI-COR www.licor.com/bio/products/software/image_studio_lite).

Supplementary Table S1: Calculated IC50 values for salinomycin treatment for human cell lines

Cell Line	IC50 (µM)	
	24 hour	48 hour
U937	25.5 ± 3.5	1.8 ± 0.5
HL60	15 ± 2.2	0.83 ± 0.1
OCI-AML3	1.38 ± 0.3	0.42 ± 0.05
K562	22.5 ± 3.4	1.65 ± 0.4
NB4	13.1 ± 1.6	0.36 ± 0.03
MOLM-13	6.3 ± 0.5	1.0 ± 0.2
NOMO-1	3.3 ± 0.4	0.75 ± 0.1
MV4;11	2.6 ± 0.6	0.55 ± 0.1
RS4;11	6.6 ± 0.6	1.4 ± 0.4

Excel spreadsheet showing the calculated IC50s for salinomycin treatment of AML and MLLr cell lines for 24 and 48 hours as measured by Cell Titre Glo™ cell viability assays.

Supplementary Table S2: Gene expression analysis of MAF9 cells. Excel spreadsheet showing absolute fluorescence intensities for 24,326 probes from the Mouse Ref-8 v.2 Illumina BeadArray normalized by quantile method using *beadarray* R package. Values for salinomycin or 0.01% DMSO treatment of MAF9 cells are presented

See Supplementary File 1

Supplementary Table S3: Salinomycin signature P<0.001. Excel spreadsheet showing the 21 Illumina probes constituting the salinomycin 17-gene signature plus Col4a1. Probes are labelled by their Illumina ID, ID symbol, ID gene name and Entrez ID. Probes are ranked based on their B-statistic (log-odds) for salinomycin treatment versus control and those with adjusted P values < 0.001 are highlighted (boxed)

See Supplementary File 2

Supplementary Table S4: Salinomycin 17-gene signature, primer sequences and amplicons

Gene Name	Primer Sequence (5′ - 3′)		Amplicon Length
	Forward	Reverse	
Ddit3	CTGGAAGCCTGGTATGAGGAT	CAGGGTCAAGAGTAGTGAAGGT	121
Gadd45a	CCGAAAGGATGGACACGGTG	TTATCGGGGTCTACGTTGAGC	121
Sqstm1	AGGATGGGGACTTGGTTGC	TCACAGATCACATTGGGGTGC	178
Col4a1	CTGGCACAAAAGGGACGAG	ACGTGGCCGAGAATTTCACC	238
Trib3	GCAAAGCGGCTGATGTCTG	AGAGTCGTGGAATGGGTATCTG	77
Mmp2	CAAGTTCCCCGGCGATGTC	TTCTGGTCAAGGTCACCTGTC	171
Ltf	TGAGGCCCTTGGACTCTGT	ACCCACTTTTCTCATCTCGTTC	112
Plekha1	CAAGGTCCAGACTGTCTCTCC	CCCTGAGGGCCGTTTTTACTC	90
Slc7a11	GGCACCGTCATCGGATCAG	CTCCACAGGCAGACCAGAAAA	100
Cxcl10	CCAAGTGCTGCCGTCATTTTC	GGCTCGCAGGGATGATTTCAA	157
Gtpbp2	GGAACAGAGGAGGTAAAGCCA	GCTCAAAGCGGTACTGAGATG	111
Herpud1	GCAGTTGGAGTGTGAGTCG	TCTGTGGATTCAGCACCCTTT	229
Ticam2	CGATCAAGACGGCCATGAGTC	CTCGTCGGTGTCATCTTCTGC	199
Nupr1	CCCTTCCCAGCAACCTCTAAA	TCTTGGTCCGACCTTTCCGA	116
Klf6	GTTTCTGCTCGGACTCCTGAT	TTCCTGGAAGATGCTACACATTG	108
Cd74	AGTGCGACGAGAACGGTAAC	CGTTGGGGAACACACACCA	78
Nfkbia	TGAAGGACGAGGAGTACGAGC	TTCGTGGATGATTGCCAAGTG	146
Ccl3	TTCTCTGTACCATGACACTCTGC	CGTGGAATCTTCCGGCTGTAG	100

Excel spreadsheet showing the named genes within the salinomycin 17-gene signature with the primer sequences and amplicon lengths for the products obtained by qRT-PCR.
